# A method to correct for local alterations in DNA copy number that bias functional genomics assays applied to antibiotic-treated bacteria

**DOI:** 10.1128/msystems.00665-23

**Published:** 2024-03-12

**Authors:** Geraldine J. Sullivan, Lars Barquist, Amy K. Cain

**Affiliations:** 1 ARC Centre of Excellence in Synthetic Biology, School of Natural Sciences, Macquarie University, Sydney, Australia; 2 Faculty of Medicine, University of Würzburg, Würzburg, Germany; 3 Helmholtz Institute for RNA-based Infection Research (HIRI), Helmholtz Center for Infection Research (HZI), Würzburg, Germany; 4 Department of Biology, University of Toronto Mississauga, Mississauga, Ontario, Canada; University of North Carolina at Charlotte, Charlotte, North Carolina, USA

**Keywords:** data normalization, transposon insertion sequencing, RNA sequencing, chromosomal copy number, bacterial genomics, ciprofloxacin, median sliding window, data correction

## Abstract

Functional genomics techniques, such as transposon insertion sequencing and RNA-sequencing, are key to studying relative differences in bacterial mutant fitness or gene expression under selective conditions. However, certain stress conditions, mutations, or antibiotics can directly interfere with DNA synthesis, resulting in systematic changes in local DNA copy numbers along the chromosome. This can lead to artifacts in sequencing-based functional genomics data when comparing antibiotic treatment to an unstressed control. Further, relative differences in gene-wise read counts may result from alterations in chromosomal replication dynamics, rather than selection or direct gene regulation. We term this artifact “chromosomal location bias” and implement a principled statistical approach to correct it by calculating local normalization factors along the chromosome. These normalization factors are then directly incorporated into statistical analyses using standard RNA-sequencing analysis methods without modifying the read counts themselves, preserving important information about the mean-variance relationship in the data. We illustrate the utility of this approach by generating and analyzing a ciprofloxacin-treated transposon insertion sequencing data set in *Escherichia coli* as a case study. We show that ciprofloxacin treatment generates chromosomal location bias in the resulting data, and we further demonstrate that failing to correct for this bias leads to false predictions of mutant drug sensitivity as measured by minimum inhibitory concentrations. We have developed an R package and user-friendly graphical Shiny application, ChromoCorrect, that detects and corrects for chromosomal bias in read count data, enabling the application of functional genomics technologies to the study of antibiotic stress.

**IMPORTANCE:**

Altered gene dosage due to changes in DNA replication has been observed under a variety of stresses with a variety of experimental techniques. However, the implications of changes in gene dosage for sequencing-based functional genomics assays are rarely considered. We present a statistically principled approach to correcting for the effect of changes in gene dosage, enabling testing for differences in the fitness effects or regulation of individual genes in the presence of confounding differences in DNA copy number. We show that failing to correct for these effects can lead to incorrect predictions of resistance phenotype when applying functional genomics assays to investigate antibiotic stress, and we provide a user-friendly application to detect and correct for changes in DNA copy number.

## INTRODUCTION

Functional genomics technologies, such as transposon insertion sequencing (TIS) (1) and RNA-sequencing (RNA-seq) (2), have emerged as effective, high-throughput methods for investigating gene function. Most analysis of functional genomics data relies on quantifying and comparing sequencing read counts, with results often expressed as relative (log) ratios of read counts between an experimental condition and control. Critically, the calculation of these log read count ratios depends on accurate normalization. Common normalization methods correct for differences in sequencing depth between experiments and assume this normalization factor is constant for all genes assayed. Here, we identify a phenomenon that violates these normalization assumptions resulting from treatments that affect DNA replication in bacteria, which we have coined chromosomal location bias (CLB). We present a new normalization technique and associated tool, named “ChromoCorrect,” that can be easily applied to any functional genomics data to identify and correct for CLB.

**Fig 1:**
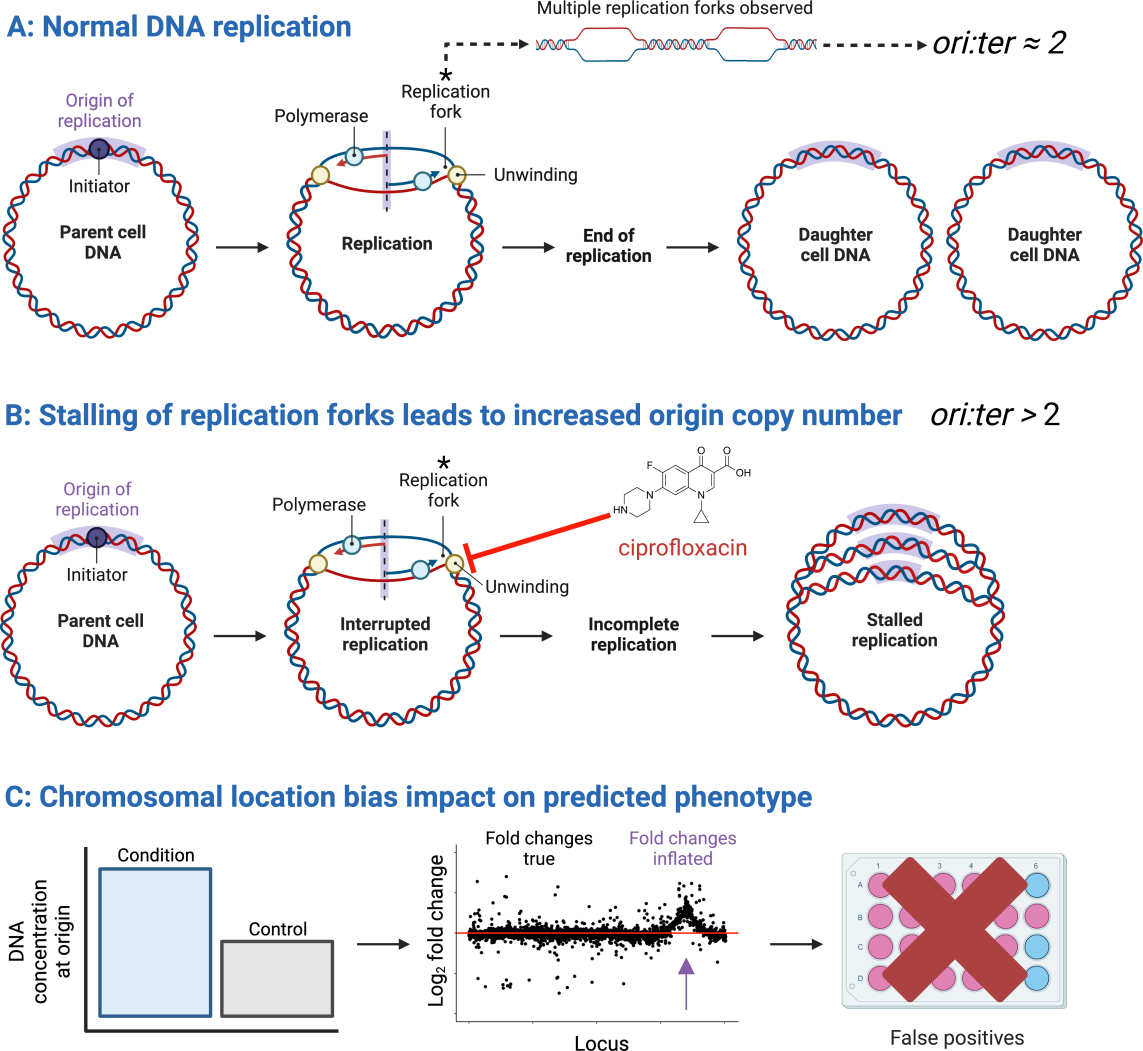
Chromosomal location bias as a result of higher chromosomal copy number near the origin after ciprofloxacin treatment and the downstream effects on read counts. **(A**) Normal DNA replication producing two daughter cells. The origin of replication is colored purple. Multiple replication forks naturally lead to more origin (*oriC*) than terminus (*ter*) DNA. **(B**) Ciprofloxacin prevents DNA unwinding, stalling the replication fork. This produces a highly inflated relative concentration of DNA proximal to the origin. **(C**) The knock-on effects of increased DNA concentration around the origin on the read counts and fold changes, an observable peak at the origin in the relative log_2_FCs and potential false positive predictions of downstream drug sensitivity testing.

During normal exponential growth in bacteria, the time between cell divisions is significantly shorter than the time needed to complete chromosomal replication. This leads to cells containing more copies of DNA around the origin of replication (*oriC*) compared to the terminus (*ter*) and is due to the firing of multiple simultaneous replication forks (3) (Fig. 1A). In many sequencing-based assays, this difference in DNA copy numbers translates into higher read counts around the origin as compared to the terminus due to a higher availability of template nucleic acids. Under most conditions, the ratio of *oriC-ter* reads remains constant between a treatment and an untreated control and does not interfere with results. However, certain treatments specifically alter the *oriC-ter* ratio, such as exposure to the DNA gyrase-targeting antibiotic ciprofloxacin (Fig. 1B). This introduces large changes in read counts that primarily reflect changes in the DNA copy number near the origin rather than gene regulation (RNA-seq) or mutant fitness (TIS) (Fig. 1C). These distortions, or CLB, in turn can lead to incorrect predictions of drug sensitivity or resistance.

A comprehensive study by Slager et al. (4) showed that treatment with a range of antimicrobials modified both DNA and RNA copy numbers, resulting in altered *oriC-ter* ratios across the genomes for multiple bacterial species. In a subsequent review, Slager et al. (5) suggested that not normalizing for this effect in RNA-seq analyses may lead to an overestimation of differential gene expression. Previous studies have attempted to correct CLB in antibiotic-treated functional genomics data using local regression methods such as Lowess (6–8). Typically, local regression is performed on the raw read counts, with normalized counts produced to replace the raw reads for the differential analysis. However, most differential analysis tools for sequencing data rely on count models that assume counts of similar magnitude have similar variance (9, 10). Providing modified or transformed counts violates these assumptions and will lead to incorrect assessment of statistical significance. We often observe distortions in the local read count density spanning several orders of magnitude following antibiotic treatment, which would lead to concomitantly large distortions in resulting *P*-values. Hence, there is a clear need for a statistically sound methodology to properly address CLB in functional genomics data.

Here, we develop a statistically principled approach for correcting CLB using a local normalization factor rather than directly providing normalized counts. These local normalization factors can then be provided to differential analysis tools such as edgeR (9) or DESeq2 (10, 11) as offsets alongside the raw counts to correct for CLB within the statistical model. This preserves important features of the data needed for accurate calculation of *P* values, namely the mean-variance trend, while also producing fold-changes that have the CLB effect removed. Based on this method we have developed an application for identifying and correcting for CLB named “ChromoCorrect.” We have made our diagnostic and normalization procedure available as a graphical Shiny application that can be applied to any sequencing-based functional genomics assay. We apply ChromoCorrect to a data set we generated for this study that displays strong CLB: TIS output of an *Escherichia coli* K12 library challenged with ciprofloxacin. We confirm that ciprofloxacin produces the predicted large local distortions in read counts around the origin of replication, confounding the TIS counts such that they no longer accurately reflect the fitness of individual mutants. We show, using minimum inhibitory concentration (MIC) assays, that these distortions lead to incorrect predictions of mutant ciprofloxacin sensitivity and resistance. We also demonstrate that our normalization approach, after processing with ChromoCorrect, corrects these, rendering accurate fold changes that align well with independently determined mutant phenotypes.

## RESULTS

### Chromosomal location bias distorts a range of functional genomics data sets

**Fig 2:**
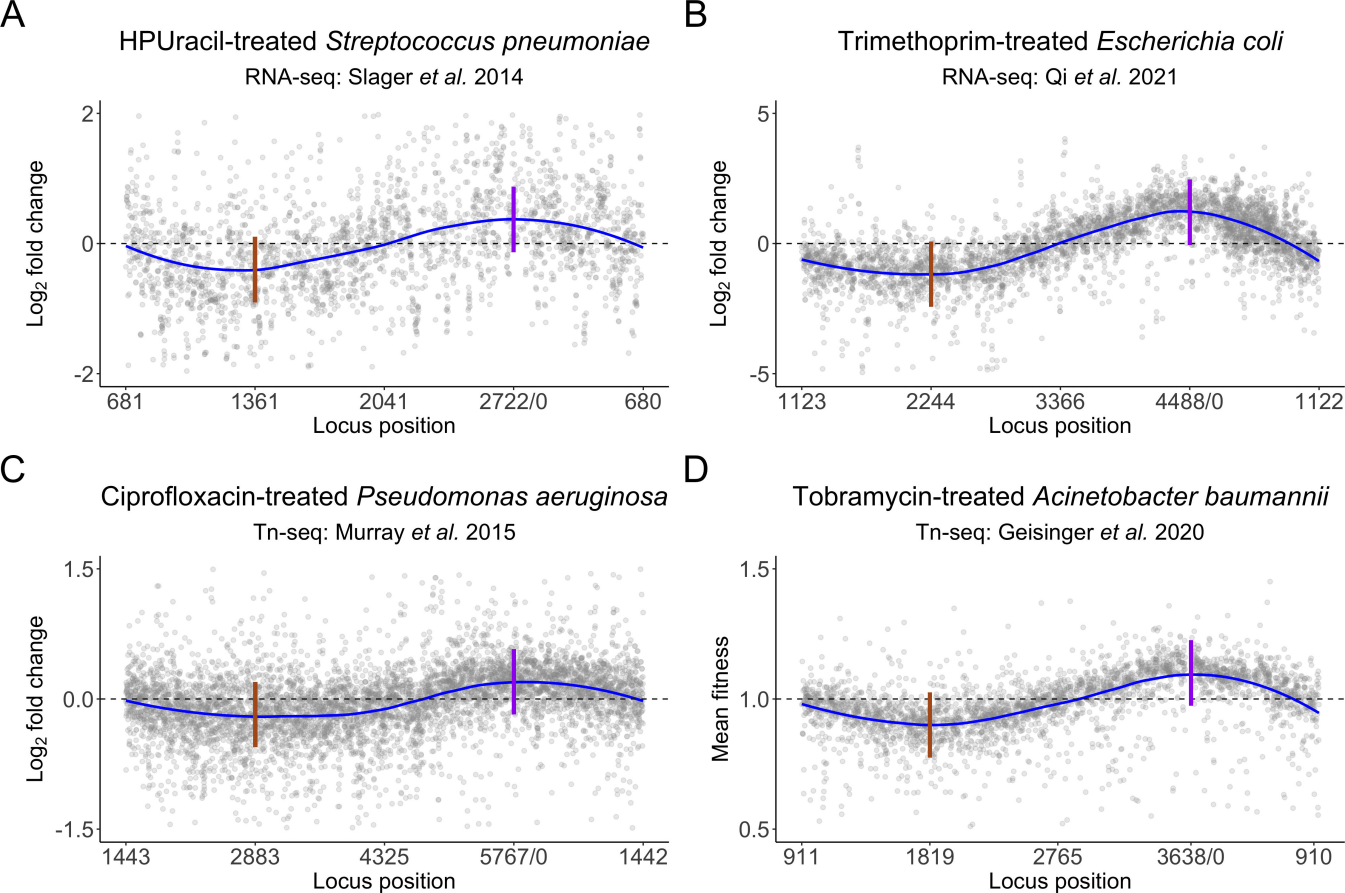
Read count log_2_ fold changes versus genome location plots displaying chromosomal location bias under varying conditions and organisms using different experimental techniques. Blue lines indicate trendlines over the individual gray points. Dashed gray lines indicate the expected trend line. Solid vertical lines indicate the terminus (brown) and origin of replication (purple). All experiments have decreased fold changes or fitness scores that dip at the terminus and increase at the origin. (**A**) RNA-Seq analysis of 6(p-Hydroxyphenylazo)-uracil-treated *Streptococcus pneumoniae*. **(B**) RNA-Seq analysis of trimethoprim-treated *Escherichia coli*. **(C**) Tn-seq analysis of ciprofloxacin-treated *Pseudomonas aeruginosa*. **(D**) Tn-seq analysis of tobramycin-treated *Acinetobacter baumannii*. Data sources: (A): Slager et al. (4), (B): Qi et al. (12), (C): Murray et al. (13), and (D): Geisinger et al. (7).

To illustrate the prevalence of CLB, we collected instances of CLB in existing RNA-seq and TIS data sets from four different antibiotic treatments across four different bacteria and plotted log_2_ fold changes along the chromosome (Fig. 2) (4, 7, 12, 13). In all cases examined, we see clear evidence of CLB, with a local increase in DNA copy number identified around the origin. Taken together, this supports that CLB is widespread in antibiotic-treated functional genomics data sets and that a robust method to detect and remove CLB is necessary to produce accurate predictions for downstream analysis.

### A principled normalization procedure to correct for chromosomal location bias

To correct CLB, we have developed a normalization procedure that produces offsets that can be directly incorporated into differential testing using packages such as edgeR (9) or DEseq2 (10) without modifying the input count data. These offsets can be thought of as a gene-specific normalization factor, which in this case includes a correction for the local read density across the chromosome.

**Fig 3:**
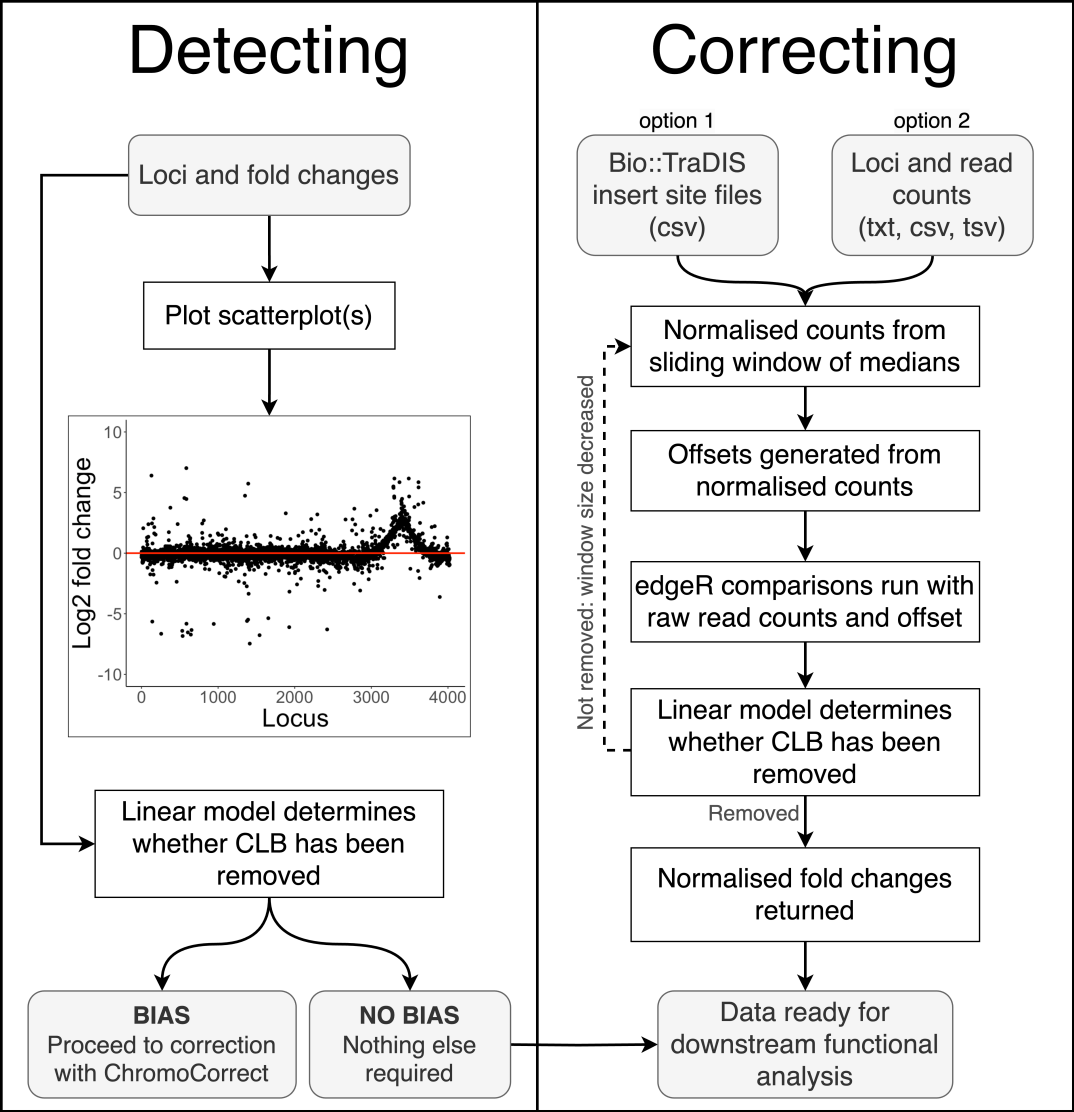
Schematic of pipeline for detecting and correcting chromosomal location bias using ChromoCorrect. Gray boxes indicate user input or output, and white indicates automated steps. Detecting requires the log_2_ fold changes of each locus to plot a scatterplot of fold change by chromosomal position, which graphs trends in the fold changes for the user to visualize the pipeline’s assessment. The app and the R console display a message recommending normalization if chromosomal location bias is detected by a fitted linear model. Correcting requires read counts per locus in a txt, csv, or tsv file format, which are then normalized using a sliding window of medians with a default size of 500. An offset matrix is generated from the normalized counts to input along with the raw read counts into edgeR. A linear model is fitted again during correction to determine whether the default sliding window is small enough to capture the trend and repeats the normalization procedure with a smaller window otherwise. The corrected analysis is returned after the normalization is complete.

Our normalization procedure comprises three major steps as outlined in Fig. 3. First, we calculate the local median read depth along the chromosome using a sliding window over the local gene neighborhood. The number of flanking genes included in the sliding window starts at 500 and is dynamically determined from the data set by fitting a linear model and testing the slope and *y*-intercept of the fitted line. Each iteration reduces the window size by 100 loci if the window is not small enough to accurately fit the trendline until a minimum window size of 200 is reached. We have found that medians calculated from windows smaller than 200 loci can be unduly influenced by a small number of genes with particularly high or low read counts. Second, the sliding window analysis is used to calculate a gene read count normalized for local read density by dividing the actual read count for each gene by the ratio of the local to global average read counts. This normalized count is then used to derive a gene-wise normalization factor, additionally incorporating differences in effective library size between replicates using the trimmed mean of *M*-values (TMM) method (14) (see Materials and Methods). Finally, these offsets are provided to the edgeR (9) glmFit function alongside the raw counts for differential analysis, where they are directly incorporated into the statistical model for testing purposes. This procedure maintains the information contained in the raw counts, necessary for accurate statistical analysis, while correcting the resulting estimated log_2_FCs for local distortions in DNA copy number.

### The ChromoCorrect app

The normalization techniques described here have been organized into an R Shiny app for easy use by researchers wanting to diagnose and normalize data affected by CLB. Instructions for installing and running the app can be found on Github (https://github.com/BarquistLab/ChromoCorrect/) or accessed online through ShinyApps (https://thecainlab.shinyapps.io/ChromoCorrect/).

### Transposon insertion sequencing case study: ciprofloxacin

**Fig 4:**
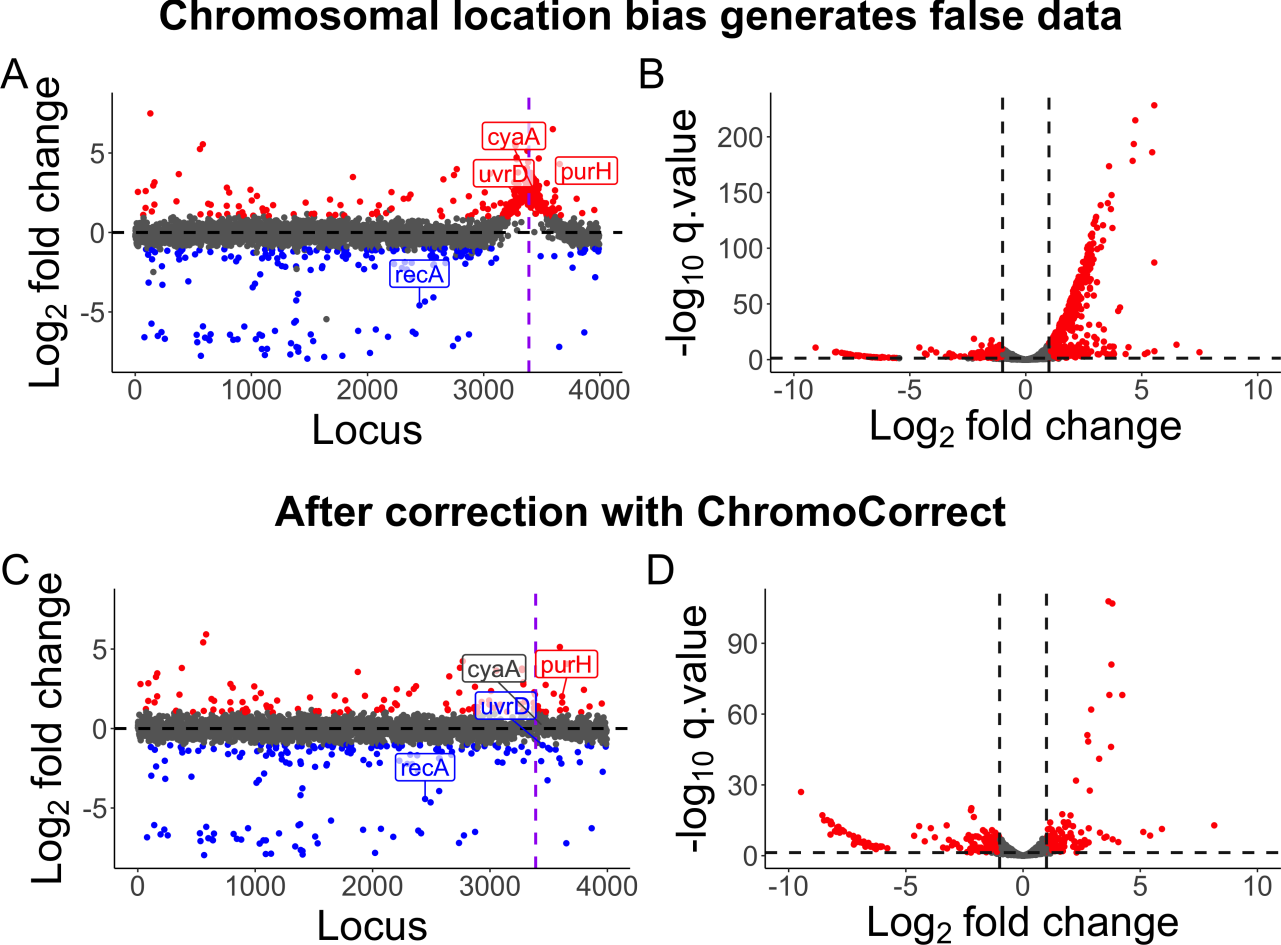
Visualizing and diagnosing chromosomal location bias in a ciprofloxacin-treated transposon insertion sequencing data set. Each point represents a locus of the genome. The *x*-axis is the chromosomal location, and the *y*-axis is the log_2_FC from ciprofloxacin treated versus untreated comparisons. **(A**) Volcano plot before correction, showing a large skew of significant genes (red) to the right, representing an increased prevalence of these mutants compared to the no antibiotic control. **(B**) Locus by fold change scatterplot pre-normalization with loci plotted in chromosomal order. The dashed black line shows the expected trend of the data if not affected by CLB. The purple dotted line is the origin of replication, where a large peak of elevated read counts is seen. **(C**) Locus by fold change scatterplot post-normalization with no peak. **(D**) Volcano plot post-normalization with no skew. Normalization performed with a sliding median window size of 200. Some mutant examples from our phenotypic validation are labeled in the scatterplots. Blue genes represent significant genes with log_2_FCs ≤ −1, and red genes indicate significant genes with log_2_FCs ≥ 1.

To illustrate the functionality of ChromoCorrect, we generated a data set applying the transposon-directed insertion-site sequencing (TraDIS) TIS technique to a dense library of *E. coli* transposon mutants exposed to ciprofloxacin compared to an untreated control. This data set was generated using *E. coli* K12 BW25113, the parental strain of the Keio collection (15). A TraDIS library of 350,000 unique Tn*5* mutants (16) was challenged with a subinhibitory concentration of ciprofloxacin (1/2 MIC) with growth overnight. After analysis with the TraDIS toolkit (17), we confirmed ciprofloxacin as an inducer of exaggerated CLB as it displayed a distinctive peak of increased reads around the origin of replication (Fig. 4A). This peak reflects the expected increase in DNA copy number at the origin compared to the rest of the genome that ciprofloxacin induces as it targets topoisomerases and stalls the replication fork and DNA synthesis (4, 18). Around the peak, the observed inflation in transposon insertions occurred largely between loci 3023 and 3622, 300 loci on either side of the origin of replication (*oriC* is located between locus 3322, *mnmG* and locus 3323, *mioC*). This entire 600 locus region had an average log_2_FC of 1.5 (a fold change increase of 2.8 compared to the untreated control), whereas the rest of the genome had a more typical average log_2_FC of 0.1. Strikingly, this means that many of these 600 loci would meet the standard “2-fold” cutoff often used to prioritize genes for further investigation.

After analysis with the TraDIS toolkit (17), the ciprofloxacin treatment TIS data yielded 754 genes with significant values (*q*-value < 0.05), with 468 having an absolute log_2_FC value over 1. Of these 468 genes, 391 (84%) of these were located between loci positions 3023 and 3623, despite this region representing only 15% of the genome. Another confirmation of this data bias was visualization by a volcano plot showing log_2_FC versus −log_10_ *q*-value (Fig. 4B). The fold change was skewed to the right, indicating a bias towards cells that appeared to have a higher frequency of insertions in many genes. After normalizing the data using ChromoCorrect with an automatically determined sliding window median of 200 genes, we identified 272 significant genes (a 64% reduction), with only 163 having an absolute log_2_FC value over 1 (a 65% reduction). The normalized volcano plot and locus by fold change scatterplot show the CLB has been removed (Fig. 3C and D).

### Chromosomal location bias leads to incorrect predictions of ciprofloxacin sensitivity

Using TIS or other functional genomics techniques to identify entire gene suites involved in antibiotic stress tolerance is well-established (8, 13, 19). However, the possible presence of CLB in these data sets, if left uncorrected, may result in false positive predictions that are carried on into laboratory analysis. To demonstrate the implications of CLB for predictions of antibiotic resistance (or sensitivity), as well as to validate the use of ChromoCorrect to generate more biologically accurate predictions, we tested the phenotypes of various *E. coli* BW25113 single gene mutants from the Keio collection (15) that represent different types of potential errors that may arise because of CLB. In total, 11 mutants were tested for their ciprofloxacin resistance and sensitivity profile (between 5 and 20 ng/mL) via an MIC assay and compared to the wild type (WT) BW25113 resistance level (10 ng/mL). For this, we examined genes that exhibited dissimilar outputs before and after correction, particularly focusing on those that were identified as significant prior to correction but not afterward. The 11 mutants tested represented four distinct classes of genes (Table 1). The first class represented a positive control and included the known antibiotic resistance determinants *acrB*, involved in efflux (20, 21), and *recA*, involved in DNA recombination and repair (22, 23). As expected, both genes remained significant after correction with ChromoCorrect with a predicted sensitivity phenotype and were confirmed by the MIC assay. The second class included five genes with a predicted sensitivity phenotype before but not after applying ChromoCorrect. As predicted by ChromoCorrect, all five mutants displayed no change in MIC compared to WT, confirming that they were falsely designated as ciprofloxacin sensitivity genes prior to correction for CLB. The third class comprised three gene representatives predicted to be sensitive both before and after correction, and all showed a twofold increase (20 ng/mL) in ciprofloxacin MIC, confirming a true sensitivity phenotype. Finally, we examined genes with the most extreme mispredictions of phenotype in the absence of correction for CLB, those that shifted from predicted mutant resistance to predicted sensitivity after correction. There were eight genes whose log_2_FC went from positive to negative values, but only one (*uvrD*) met the phenotypic validation threshold of |log_2_FC| ≥ 1 before and after correction. The misprediction of *∆uvrD* phenotype prior to correction was confirmed by increased ciprofloxacin sensitivity in our MIC assay. Neglecting to address this bias would have falsely classified *uvrD* as mediating ciprofloxacin sensitivity, not resistance. In summary, all mutants tested by MIC assay reflected the predicted phenotype after correction of CLB, including six instances where the analysis based on uncorrected data led to an incorrect prediction of resistance phenotype. This emphasizes the critical role of ChromoCorrect’s normalization in ensuring accurate and reliable gene fitness assessments.

**TABLE 1:** Single gene *E. coli* BW25113 Keio knockouts validated in this study

Predicted mutant phenotype	Gene	Function	TraDIS log_2_FC		Experimental mutant phenotype MIC_50_ (ng/mL)
			Before ChromoCorrect	After ChromoCorrect	
-	WT	-	-	-	10
Cip sensitive before and after	*acrB*	Multidrug efflux system protein	−0.89	−0.83	≤5
	*recA*	DNA recombination and repair protein	−1.99	−1.92	≤5
Cip resistant before and WT phenotype after	*cyaA*	Adenylate cyclase	2.48	0.15	10
	*ilvN*	Acetolactate synthase 1 small subunit	2.13	−0.06	10
	*mtlD*	Mannitol-1-phosphate dehydrogenase	1.77	0.13	10
	*fdhE*	Formate dehydrogenase formation protein	1.66	0.13	10
	*pfkA*	6-phosphofructokinase I	1.32	−0.03	10
Cip resistant before and after	*purH*	Bifunctional transformylase/ cyclohydrolase	2.50	1.64	20
	*dgkA*	Diacylglycerol kinase	1.62	1.07	20
	*rpe*	D-ribulose-5-phosphate 3-epimerase	1.45	1.07	20
Cip resistant before and sensitive after	*uvrD*	DNA-dependent ATPase I and helicase II	1.32	−1.06	≤5

### Use of the Shiny application

The app is split into two main tabs: *detecting* and *correcting* (Fig. 3). *Detecting* requires the upload of analyzed output files containing log_2_FC values to visualize any CLB, while the *Correcting* tab requires the upload of the read counts for the conditions affected by CLB and the associated no-stress control.

The first step within the app is to assess whether data sets that have undergone differential analysis are affected by CLB. This can be done using the *Detecting* tab. The user inputs one or more files containing locus tags and fold change information, and a locus by fold change scatterplot for each condition is generated. The user can cycle through the uploaded data sets in the drop-down menu in the sidebar to determine if any of the experimental conditions are affected by CLB. It is deemed present if the general trend of the fold changes is not flat and distributed around zero, as demonstrated in Fig. 3A. The “decision” box will contain red text suggesting correction if CLB is detected, or black text if not detected.

If the analysis produced CLB, the user can fix the issue with the second tab: *Correcting*. This tab requires the upload of a file with a locus_tag column and read counts, or the upload of read files produced by the tradis_gene_insert_sites script from Bio-TraDIS (17). The tab requires at least four insert site files or columns of read counts: two biological replicates of the experimental condition and two biological replicates of the associated control condition. Once the upload is complete, the user must define which condition specifies the control. The app will compute an edgeR comparison before and after normalization and produce two scatterplots of locus versus log fold change associated with the two analyses. The default window size of 500 is suitable for smooth trend lines. The window will automatically reduce in size if the data has not been normalized effectively due to any sharper trend lines present. The user can then export the corrected output, free from CLB.

## DISCUSSION

This study addresses the fundamental issue of CLB, which we show impacts a wide variety of functional genomics analyses, resulting in false positives and negatives or incorrect interpretation of data. CLB arises from nucleic acid copy number fluctuations along the chromosome, typically around the origin and/or terminus, which can be exacerbated by replication-disrupting events like replication-targeted antibiotic treatment. To solve this problem, we introduce ChromoCorrect, a normalization tool that effectively corrects for CLB producing accurate log_2_FCs and significance values for each locus. Using a ciprofloxacin-treated TraDIS data set in *E. coli*, we demonstrated that CLB leads to incorrect predictions of antibiotic resistance phenotypes that can be corrected using ChromoCorrect.

Over the past decade, functional genomics techniques, like TIS and RNA-Seq, have been employed to comprehensively assess the effects of various selection pressures, such as antibiotic exposure, on microbial fitness and cellular responses (8, 13, 19, 24, 25). Here, we show that CLB can lead to incorrect prediction of phenotype using our own antibiotic-treated TIS data, as a cautionary tale for future studies. Our study retrospectively highlighted the prevalence of CLB in published functional genomics data sets across diverse species and under a range of conditions. These conditions include both treatment with direct DNA-targeting antimicrobials, such as fluoroquinolones, but also antimicrobials that have an indirect effect on DNA replication, like trimethoprim, HPUra, and tobramycin (Fig. 2) (4, 7, 12, 13). Trimethoprim targets the dihydrofolate reductase of the folate biosynthesis pathway and reduces the availability of tetrahydrofolate, a precursor to the essential DNA components thymidine and thymine (26) eventually leading to “thymineless death” (27). Thymine starvation stalls replication forks, initially leading to a transient increase in origin-proximal DNA before the replicating DNA is destabilized and degraded, leading to ultimate depletion of origin-proximal DNA (28). Similarly, the uracil analogue HPUra has an indirect effect on DNA replication by stalling replication forks (4). The mechanism by which tobramycin, a ribosome-targeting antibiotic, contributes to the observed CLB is not obvious but could represent another indirect or secondary effect. Our work suggests that CLB may be exerting a more significant influence on the interpretation of sequencing data than currently acknowledged and an important future study would be to comprehensively assess the prevalence of CLB across functional genomics data sets.

The four organisms we investigated span both Gram-negative and Gram-positive bacteria, indicating that CLB occurs in diverse species, and is likely relevant beyond these well-studied organisms. Our method bears conceptual similarity to peak-to-trough ratio (PTR) methods that use *oriC-ter* ratios to determine bacterial growth rates from genomic or metagenomic DNA sequencing data (29–31), which have been shown to accurately predict growth rates in a wide range of bacteria in pure culture. The success of these methods suggests that beyond antibiotic treatments that interfere with DNA replication, CLB may also affect the results of functional genomics comparison between bacterial cultures growing at different rates.

Biases arising from altered *oriC-ter* ratios in TIS data have been recognized previously and corrected for using local regression methods (6–8), though the origins of these biases have not been clearly described. Our contribution has been to highlight the prevalence of CLB in bacterial functional genomics data. A major advantage of ChromoCorrect is an ability to directly incorporate the local normalization factors as an offset into differential analysis tools such as edgeR (9) and DESeq2 (10). This preserves the mean-variance relationship in the underlying count data, which is important for accurate estimation of statistical significance. This approach was inspired by the transcript quantification package tximport that corrects for differences in isoform abundance during gene-level differential expression in eukaryotes (32). By facilitating the import of offsets into established RNA-seq analysis tools, ChromoCorrect can be used seamlessly with existing pipelines.

Although clearly a critical step in the analysis of functional genomics data, the interpretation of correcting for CLB requires care and depends on the technology analyzed. In the case of RNA-seq, CLB is reflective of a genuine increase in RNA synthesized from the origin-proximal region, and the primary danger is that this could be interpreted as a specific regulatory response rather than a direct result of antibiotic activity on DNA replication dynamics. In contrast for TIS experiments, CLB introduces artifacts that can lead to false predictions of phenotype. Since TIS uses transposon-flanking reads as a proxy for mutant abundance, and local distortions in DNA copy number will lead to a local distortion in template DNA abundance, mutants containing transposon insertions in the vicinity of the origin will appear to be more frequent in the population than they really are in data affected by CLB.

Our study highlights the importance of scrutinizing data for CLB to improve the reliability of conclusions drawn from functional genomics data. We recommend that future microbial functional genomic data sets with read counts produced, especially those that involve antibiotic exposure, be screened for the presence of CLB, and if so, to correct the data using ChromoCorrect before proceeding with time and labor-intensive biological interpretation and laboratory experiments.

## MATERIALS AND METHODS

### Software

Analyses were performed using R (version 4.0.3), R Studio (version 2022.07.2). The application was developed using RShiny (version 1.7.3).

### TraDIS library ciprofloxacin challenging and sequencing

An *E. coli* K12 TraDIS library was generated as previously described (16) and challenged with subinhibitory ciprofloxacin (40 µg/mL) in 10 mL of Mueller Hinton cation-adjusted media. Genomic DNA was extracted using the DNeasy UltraClean Microbial Kit (Qiagen) according to the manufacturer’s instructions and was sequenced on an Illumina HiSeq2500 platform at the Wellcome Sanger Institute.

### Identifying chromosomal location bias

The data were run through the Bio::TraDIS pipeline (17) using SMALT mapping and a minimum read count of 10. To identify whether CLB was present, the log_2_FCs were plotted in genome order, with locus on the *x*-axis and log_2_FC on the *y*-axis.

### Generating normalized read counts and offsets with ChromoCorrect

For each condition, the read counts are obtained, and the first 1,000 genes are appended to the end of the file and the last 1,000 to the beginning to mimic a circular genome for the median sliding window function. Read counts of zero are excluded from the data set to remove their influence on the median. The medianFilter function from package FBN (version 1.5.1) is used to calculate a median for each locus based on an adjustable window size. We have found that a default window size of 500 is sufficient for many smoother trends, whilst sharps trends need to be computed with a smaller window size. A ratio for each point is calculated by dividing the locus’ median by the mean median of all loci. The normalized read count is obtained by dividing the raw read count by the ratio computed for each locus. The normalized counts are not used as a replacement for the raw read counts, instead, an offset data set is created. The offset is computed as the natural logarithm of the raw count, subtracted from the natural logarithm of the normalized count. An arbitrary pseudocount of 0.1 is added to both raw and normalized counts before log transforming to prevent the undefined log of 0 occurring. The effective library size is calculated using the edgeR package (version 3.32.1) calcNormFactors function applied to the normalized counts, multiplied by the column sums of the normalized counts. Following this, the offset values are adjusted to account for library size differences by subtracting the logarithm of the effective library size.

### Comparisons using edgeR within ChromoCorrect

ChromoCorrect incorporates the offset along with the raw counts in edgeR to perform differential analysis and assess the normalization. Genes are filtered based on a minimum count threshold (default value of 10). Raw counts are put into a DGEList with groupings and scaleOffset() is used to offset the raw read counts. Library size normalization is not computed due to the inclusion of the offset, which produces a custom normalization factor per gene. Common negative binomial (estimateGLMCommonDisp()) and Bayes tagwise (estimateGLMTagwiseDisp()) dispersions for general linear models are calculated. A gene-wise negative binomial for general linear models (glmFit() and glmLRT()) is fit with contrasts to produce likelihood ratio tests per gene between the control and conditions, producing the log_2_ fold changes and adjusted *P* values. Following the analysis, ChromoCorrect generates summary statistics and diagnostic plots, automatically calculating the adjustment of the window size if bias has not been mitigated. The process repeats until a satisfactory result is achieved or a minimum window size of 200 is reached. The code produces scatterplots of the data before and after bias is removed for a visual reference.

### Minimum inhibitory concentration assays

MIC assays were performed for the single gene knockouts to determine their breakpoint compared to WT cultures. Mutants were steaked from frozen onto Mueller Hinton (MH) agar plates and incubated overnight at 37˚C. Three single colonies of each mutant were inoculated into 5 mL of cation-adjusted MH broth (CAMHB) and grown overnight at 37˚C and 200 rpm shaking. A 1/100 dilution of the overnight cultures was made in 5 mL of fresh CAMHB and grown for 2.5 h until the exponential phase. MICs were performed with triplicate technical replicates in a 96-well plate with approximately 1 × 10^5^ cells per 150 µL well and grown overnight at 37˚C with 200 rpm shaking. Cells were imaged after 16 h at OD_600_ on a PHERAstar plate reader (BMG Labtech). Wells were blanked and averaged within triplicates. MIC was determined as the lowest concentration that inhibited at least 50% of growth compared to the untreated mutant positive control.

## Data Availability

TraDIS sequencing reads were deposited in the European Nucleotide Archive (ENA) under study accession number PRJEB35059. Control conditions are biosamples SAMEA6429763 and SAMEA6429764, ciprofloxacin conditions are SAMEA6429767 and SAMEA6429768. RNA sequencing data in S. pneumoniae reported in Slager et al. (4) sourced from ENA study accession PRJNA235855. RNA-Seq data in P. aeruginosa reported in Murray et al. (13) sourced from ENA study accession PRJNA291292. The ChromoCorrect code is publicly available and can be found on Github at https://github.com/BarquistLab/ChromoCorrect and as an online interface at https://thecainlab.shinyapps.io/ChromoCorrect/.
